# Ancient Origins of RGK Protein Function: Modulation of Voltage-Gated Calcium Channels Preceded the Protostome and Deuterostome Split

**DOI:** 10.1371/journal.pone.0100694

**Published:** 2014-07-03

**Authors:** Henry L. Puhl, Van B. Lu, Yu-Jin Won, Yehezkel Sasson, Joel A. Hirsch, Fumihito Ono, Stephen R. Ikeda

**Affiliations:** 1 Laboratory of Molecular Physiology, Section on Transmitter Signaling, National Institute on Alcohol Abuse and Alcoholism, National Institutes of Health, Rockville, Maryland, United States of America; 2 Laboratory of Molecular Physiology, Section on Model Synaptic Systems, National Institute on Alcohol Abuse and Alcoholism, National Institutes of Health, Rockville, Maryland, United States of America; 3 Department of Biochemistry & Molecular Biology, Faculty of Life Sciences, Institute for Structural Biology, Tel Aviv University, Ramat Aviv, Israel; Vanderbilt University Medical Center, United States of America

## Abstract

RGK proteins, Gem, Rad, Rem1, and Rem2, are members of the Ras superfamily of small GTP-binding proteins that interact with Ca^2+^ channel β subunits to modify voltage-gated Ca^2+^ channel function. In addition, RGK proteins affect several cellular processes such as cytoskeletal rearrangement, neuronal dendritic complexity, and synapse formation. To probe the phylogenetic origins of RGK protein–Ca^2+^ channel interactions, we identified potential RGK-like protein homologs in genomes for genetically diverse organisms from both the deuterostome and protostome animal superphyla. RGK-like protein homologs cloned from *Danio rerio* (zebrafish) and *Drosophila melanogaster* (fruit flies) expressed in mammalian sympathetic neurons decreased Ca^2+^ current density as reported for expression of mammalian RGK proteins. Sequence alignments from evolutionarily diverse organisms spanning the protostome/deuterostome divide revealed conservation of residues within the RGK G-domain involved in RGK protein – Ca_v_β subunit interaction. In addition, the C-terminal eleven residues were highly conserved and constituted a signature sequence unique to RGK proteins but of unknown function. Taken together, these data suggest that RGK proteins, and the ability to modify Ca^2+^ channel function, arose from an ancestor predating the protostomes split from deuterostomes approximately 550 million years ago.

## Introduction

The RGK protein family, comprised of Gem, Rad, Rem1, and Rem2, is an atypical subset of the Ras superfamily [1] of small GTP-binding proteins. Although the founding RGK members, Rad and Gem, were discovered over 20 years ago [2]–[4], the physiological functions of RGK proteins remain unclear. Rad was initially cloned based on differential mRNA expression in type II diabetic muscle [2]. Likewise, Gem (also known as *kir*) was cloned based on differential expression from mitogen-induced T-lymphocytes [3] and *abl* tyrosine kinase oncogene transformed hematopoietic cells [2]. Rem1 [5] and Rem2 [6] were later discovered based on sequence homology to the existing RGK proteins.

The connection between RGK proteins and voltage-gated Ca^2+^ channel (VGCC) function arose from a yeast-two hybrid screen for protein partners that interact with VGCC β-subunits (Ca_v_β). Béguin et al. [7] demonstrated that Gem bound directly to Ca_v_β and inhibited VGCC function by interfering with trafficking of high-voltage activated Ca^2+^ channels (i.e., those containing Ca_v_β) to the plasma membrane. Subsequent studies confirmed and extended these observations demonstrating that all four RGK family members interacted with all four Ca_v_β subunits (*CACNB1-4*) to attenuate VGCC function [8]-[11]. Additional mechanisms underlying attenuation of VGCC have been proposed including production of a non-conducting species [12]–[14], disruption of channel gating [15], and binding to the α_1_-subunit of some VGCCs [16]. Genetic ablation of Rem [17] or Rad [18] in mice results in increased L-type Ca^2+^ current in cardiac myocytes suggesting that endogenous RGK proteins tonically inhibit VGCC function in this tissue.

Additional functions for RGK proteins have emerged from large-scale genomic screens. For example, Rem2 emerged from an RNAi screen designed to identify proteins involved in CNS synaptogenesis [19]. Subsequent work confirmed that Rem2 impacts synapse development and dendritic morphology [20], [21]. Likewise, Gem may impact dendritic arborization and participate in certain forms of autism [22]. In non-mammalian vertebrates, RGK proteins also influence neuronal development. Inhibition of Rem2 expression in embryonic *Danio rerio* (zebrafish) interfered with midbrain development at 36 hours post fertilization [23]. Finally, a differential display screen in the urodele amphibian *Cynops pyrrhogaster* (Japanese fire belly newt) to identify proteins involved in limb regeneration revealed that Rad was highly up-regulated in skeletal muscle at the site of amputation [24]. Together, these indicate that: 1) RGK proteins serve important functions during development or regeneration of excitable cells, and 2) orthologs of RGK proteins are found throughout the vertebrate lineage.

Given the multitude of pathways in which RGK proteins participate, the question of whether RGK protein–VGCCs interactions are integral to function has arisen [21], [25]. To address this, we sought to determine whether modulation of VGCC function and residues involved with Ca_v_β interaction were conserved in evolutionarily diverse organisms. Here, we identify and clone RGK and RGK-like proteins from *Danio rerio* and *Drosophila melanogaster* and show that expression in mammalian neurons attenuates VGCC current density. Identification of RGK protein homologs in non-vertebrate deuterostomes and multiple protostome phyla followed by sequence comparison revealed that residues important for Ca_v_β binding were highly conserved. In addition, highly conserved residues specific to RGK proteins were identified. These data suggest that RGK protein interactions with VGCC functions arose in a common ancestor predating the protostome-deuterostome split occurring around 550 million years ago.

## Materials and Methods

### Electrophysiology

#### Superior cervical ganglion (SCG) neuron dissociation and intranuclear microinjection of cDNA

All animal studies were conducted in accordance with the National Institutes of Health's Guidelines for Animal Care and Use and approved by the National Institute on Alcohol Abuse and Alcoholism Animal Care and Use Committee. SCG neurons from adult (6–12 week old) Wistar rats were dissected and dissociated as described previously [26], [27]. Briefly, animals were anesthetized by CO_2_ inhalation and decapitated before dissection. Two SCG per rat were removed, de-sheathed, cut into small pieces, and incubated in modified Earles' balanced salt solution (EBSS) containing 2 mg/mL collagenase (CLS4; Worthington Biochemical, Lakewood, NJ), 0.6 mg/mL trypsin (Worthington Biochemical) and 0.1 mg/mL DNase I at 36°C for 1 hour in a water bath shaker rotating at 110 rpm. The EBSS was supplemented with 3.6 g/L d-glucose and 10 mM HEPES. After incubation, neurons were mechanically dissociated by vigorously shaking the flask for 10 s. Neurons were centrifuged at 50×g for 6 min and resuspended in Minimal Essential Media (MEM) with 10% fetal calf serum twice before being plated on poly-l-lysine coated tissue culture dishes. Cells were maintained in a humidified 95% air/5% CO_2_ incubator at 37°C.

Three to six hours after dissociation, plasmid constructs were injected directly into the nucleus of SCG neurons as described previously [26]–[28]. Briefly, cDNA was injected with a FemtoJet microinjector and 5171 micromanipulator (both from Eppendorf, Hauppauge, NY) using an injection pressure and duration of 140–160 hPa and 0.3 s, respectively. Injected plasmids were diluted in EB buffer (10 mM Tris-HCl, pH 8.5) and centrifuged in capillary tubes at 10,000 rpm for at least 30 min to separate particulate contaminants in the cDNA preparation. Cloned *Danio rerio* RGK protein ortholog and *Drosophila melanogaster* RGK-like protein homolog cDNA constructs were injected at 50–100 ng/µl together with pEGFP cDNA (Clontech, Mountain View, CA) at 5 ng/µl to identify successfully injected neurons. Following injections, neurons were incubated overnight at 37°C and electrophysiological experiments were performed the following day.

### Electrophysiology

Ca^2+^-channel currents (*I_Ca_*) were recorded using a patch-clamp amplifier (Axopatch 200B, Molecular Devices, Sunnyvale, CA) and conventional whole-cell patch-clamp techniques [29]. Patch electrodes were pulled from borosilicate glass capillaries (1.65 mm outer diameter, 1.20 mm inner diameter, King Precision Glass, Claremont, CA) using a Model P-97 micropipette puller (Sutter Instrument, Novato, CA). The patch electrodes were coated with silicone elastomer (Sylgard 184, Dow Corning, Midland, MI) and fire-polished. A Ag/AgCl pellet connected to the bath solution via a 0.15 M NaCl/agar bridge was used as a ground. Voltage protocol generation and data acquisition were performed using custom-designed software (S5) on a Macintosh G4 computer (Apple, Cupertino, CA). Current traces were filtered at 2 kHz (−3 dB; 4-pole Bessel), digitized at 10 kHz with a 16-bit analog-to-digital converter board (ITC-18, HEKA, Bellmore, NY) and stored on the computer for later analyses. Uncompensated cell capacitive currents were elicited by a 5 ms, +10 mV step from a holding potential of −80 mV immediately after establishment of the whole-cell configuration. *I_Ca_* traces were recorded following cancellation of cell membrane capacitance and series resistance compensation (>85% prediction and correction; lag set to 5 µs).

Pipette solution contained (in mM) 120 N-methyl-d-glucamine, 20 tetraethylammonium hydroxide (TEA-OH), 11 EGTA, 10 HEPES, 10 sucrose, 1 CaCl_2_, 14 Tris-creatine phosphate, 4 MgATP and 0.3 Na_2_GTP, pH 7.2 with methanesulfonic acid. External *I_Ca_* recording solution consisted of (in mM) 140 methanesulfonic acid, 145 TEA-OH, 10 HEPES, 10 glucose, 10 CaCl_2_ and 0.0003 tetrodotoxin (TTX; Alomone Laboratories, Jerusalem, Israel), pH 7.4 with TEA-OH.

### Data analysis and statistical testing

Igor Pro version 6 (WaveMetrics, Portland, OR) was used to analyze current traces. Cell membrane capacitance was calculated from uncompensated capacitive current recordings using the equation *C_m_*  =  *Q/V* where *C_m_* is the cell membrane capacitance (in pF), *Q* is the charge stored in the capacitor/cell membrane (in coulombs, derived from integrating the area under the capacitive transient current) and *V* is the amplitude of the voltage step (in volts). *I_Ca_* amplitude was measured isochronally 10 ms after the initiation of the voltage step. *I_Ca_* density (pA/pF) was calculated by dividing *I_Ca_* amplitude by *C_m_*.

Statistical tests were performed with Prism 6 for Mac OS X (GraphPad Software, La Jolla, CA). All data were expressed as mean ± SEM. Statistical significance between two groups was determined using an unpaired Student's *t* tests. Comparisons of multiple groups against a pooled control was done using one-way analysis of variance (ANOVA) followed by a Dunnett's post-test. *P<0.05* was considered statistically significant.

### Molecular Biology

Zebrafish RGK protein orthologs were cloned from cDNA prepared from an adult male fish using the RNeasy kit (Qiagen, Germantown, MD). Briefly, after anesthesia with MS-222 (tricaine methanesulfonate), a section (approximately 50 mg) was dissected from the trunk immediately caudal to the gills. The section was ground with a small ground glass pestle in a 1.5 ml microcentrifuge tube containing 600 µl of RTL buffer. The lysate was applied to a QiaShredder column to shear genomic DNA. The resulting lysate (approximately 450 µl) was used to isolate total RNA as per the RNeasy kit instructions. Total RNA concentration was determined from absorbance at 260 nm. First strand cDNA was generated from 1 µg of total RNA using the RT for PCR kit (PT1107-1) from Clontech and oligo dT primer.

Primers for the amplification of zebrafish RGK protein orthologs were designed based on the following reference sequences: Gem (NM_001045849), Rad (NM_199798), Rem1 (NM_201174), and Rem2 (NM_001123046). All four sets were designed to incorporate *MluI* or *NotI* sites (bolded and underlined) on the 5′ or 3' ends of the open reading frames, respectively. The PCR amplification was performed using *PfuUltra* Hotstart polymerase from Agilent Technologies (Santa Clara, CA). The products were cloned into the pCI vector (Promega, Madison, WI) and sequence verified. Primer sequences were as follows:

*dr_Gem* for: GATC**ACGCGT**ACCATGACCCTGCTGGCGAGCGTGC

*dr_Gem* rev: GATCGATC**GCGGCCGC**TTACAGACTCATCAGGTCATGAC

*dr_Rad* for: GATC**ACGCGT**ACCATGACTTTGAACAAAGGAGACAAG

*dr_Rad* rev: GATCGATC**GCGGCCGC**TTATAGCACTGAAAGGTCGTGGC

*dr_Rem1* for: GATC**ACGCGT**ACCATGACACTCAACACACAGAAGG

*dr_Rem1* rev: GATCGATC**GCGGCCGC**TCACAGCACAGCGAGGTCATGG

*dr_Rem2* for: GATC**ACGCGT**ACCATGTCGGACCAGGGTTATGGC

*dr_Rem2* rev: GATCGATC**GCGGCCGC**TCACATTAAAGCGCTGAGGTCG

*Drosophila melanogaster* RGK-like protein homologs were cloned from adult polyA+ RNA (Clontech). One microgram of PolyA+ RNA was used with the RT for PCR kit to generate first strand cDNA as above. Primers for the amplification of *Drosophila melanogaster* RGK proteins were designed based on the following sequences: RGK1 (AAF57577), RGK2 (AAF57577) and RGK3 (ABV53867). All three sets were designed to incorporate *MluI* or *NotI* sites (underlined) on the 5′ or 3′ ends of the open reading frames, respectively. The PCR amplification was performed using *PfuUltra* Hotstart polymerase. The products were cloned into the pCI vector and sequence verified. Primer sequences were as follows:

*dm_RGK1* for: GATC**ACGCGT**ACCATGGCGCCCTTCTACAAGCGC

*dm_RGK1* rev: GATCGATC**GCGGCCGC**TTAGAGTACATGCAGATTCTCGC

*dm_RGK2* for: GATC**ACGCGT**ACCATGGCCCAGCAACAGCGCAGC

*dm_RGK2* rev: GATCGATC**GCGGCCGC**TTATAGCACATGCAGATTCTCG

*dm_RGK3* for: GATC**ACGCGT**ACCATGGTGGACGACATCTCACCG

*dm_RGK3* rev: GATCGATC**GCGGCCGC**TTAGAGCACCTGCAGATTCTCGC

A second RGK2 forward primer was designed to amplify from the second methionine residue after several attempts to generate a full length predicted product failed. This sequence is designated *dm_RGK2t* in the text.

*dm_RGK2t* for: GATC**ACGCGT**ACCATGGCCCAGCAACAGCGCAGC

The mouse DiRas2 open reading frame (NM_001024474) was amplified from mouse whole brain cDNA and cloned into the *MluI* and *NotI* sites of pCI (Promega). The open reading frame for the fluorescent protein variant Venus was amplified with *MluI* sites on both ends and cloned into the *MluI* site of the DiRas2 pCI clone. The insertion orientation was confirmed by sequencing. Open reading frames for human Rit1 (AF084462) and Rit2 (NM_002930) were amplified from human whole brain cDNA (Clontech) and cloned into pcDNA3.1 at the *KpnI*/*XhoI* sites for Rit1, or the *BamHI*/*XhoI* sites for Rit2. All clones were sequence verified.

### RGK protein ortholog search strategies

Initial identification of RGK protein orthologs/homologs utilized standard tools such as pre-computed NCBI (http://www.ncbi.nlm.nih.gov) BlastP searches (BLink), annotated databases such as *Ensembl* (http://www.ensembl.org/) gene trees and NCBI UniGene, and individual BlastP [30] searches using EnsemblMetazoa (http://metazoa.ensembl.org/) for non-vertebrate species using human RGK protein sequences as the query. After examining the sequence of several protostome RGK-like protein homologs, we generated a search protocol using the last eleven amino acids as PHI-BLAST pattern and human Gem or Rad as the query sequence: [KR][SAF][KR][SH][C][HNED][DNEV][LM]x[VSA][L]. The PHI-BLAST[31] search set was the non-redundant protein sequences database (nr). The algorithm parameters were the defaults with the exception of the maximum target sequences which was increased to 1000. This was followed by a 2^nd^ iteration PSI-BLAST. Protostome RGK orthologs were found together with vertebrate Ras superfamily members such as Rit1 and Rit2 in the hit list as sequence identity decreased.

## Results

### Cloning and expression of zebrafish RGK protein orthologs

Orthologs of RGK proteins within the vertebrate lineage were easily identified using the NCBI Basic Alignment Search Tool (BLAST) using human RGK proteins as the search query. At present, an *Ensembl* analysis of human Gem identifies 56 orthologs in vertebrates and expansion of the pre-computed genetic tree reveals 217 genes that represent the four known family members from mammals to ray-finned fish. We decided to clone and express RGK proteins identified in *Danio rerio* for several reasons. First, genes encoding putative orthologs of Gem, Rad, Rem1, and Rem2 were readily identified in the *Danio rerio* genome. Second, transcripts for some RGK proteins have been verified and their function inferred from knockdown or screening studies [23], [32], [33]. Third, functional expression of zebrafish cDNAs in mammalian cell lines has been demonstrated [34]. Finally, the popularity of zebrafish as a vertebrate model organism results in a more refined (high coverage and better annotation) genomic database and easily obtainable mRNA/cDNA for cloning.

PCR amplification of predicted open-reading frames from *Danio rerio* cDNA produced full length clones denoted *dr_Gem*, *dr_Rad*, *dr_Rem1*, and *dr_Rem2* (NP_001039314, NP_956092, NP_957468, and NP_001116518, respectively). Within the conserved Ras-homology or G-domain, amino acid similarity with human orthologs was 80–90% except for *dr_Rem2* which was 54%. To explore the functional consequences of *Danio rerio* RGK proteins, mammalian expression vectors containing the cloned open-reading frames were injected into adult rat sympathetic ganglion neurons and the impact of the resulting expression on *I_Ca_* properties assayed as previously reported [12], [35]. The predominant *I_Ca_* component in rat SCG neurons arises from Ca_V_2.2 or ω-conotoxin GVIA-sensitive N-type channels [36]. Unlike co-expression experiments (i.e., RGK protein and VGCC subunits expressed simultaneously) in mammalian clonal cells or *Xenopus* oocytes, the native VGCC in adult sympathetic neurons are present prior to RGK protein overexpression and thus less influenced by forward trafficking perturbation [12].

**Figure 1:**
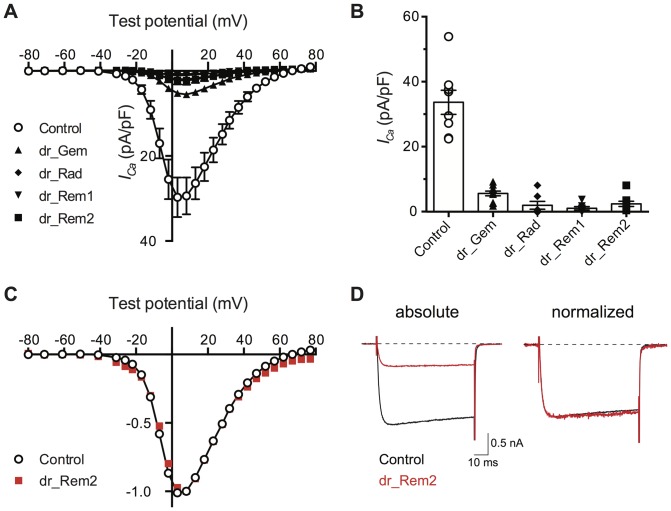
Heterologous expression of zebrafish RGK protein orthologs reduces *I_Ca_* density in rat sympathetic neurons. A. Current-voltage (*I-V*) plots in which mean ± SEM *I_Ca_* density (pA/pF) is plotted versus test potential (mV). *I_Ca_* was evoked from a holding potential of −80 mV to the test potentials indicated. *I_Ca_* amplitude, determined 10 ms after initiation of the test pulse, was normalized to membrane capacitance (*C_m_*). Control neurons (open circles) were injected with EGFP cDNA (n = 6). Neurons previously injected with *Danio rerio* RGK protein cDNA clones (100 ng/µl approximately 18–24 hours prior to recording) are depicted with filled symbols: *dr_Gem* (triangle, n = 11); *dr_Rad* (diamond, n = 7), *dr_Rem1* (inverted triangle, n = 7), and *dr_Rem2* (square, n = 9). B. Category plot of data shown in panel A for *I_Ca_* density at +10 mV. Mean *I_Ca_* density for neurons expressing RGK protein clones differed significantly (*P*<0.05) from control (one-way ANOVA followed by Dunnett's multiple comparisons test). C. Normalized *I-V* plots for control (open circle) and *dr_Rem2* expressing (red filled square) neurons. Data, from panel A, was normalized to the maximal *I_Ca_* density and plotted to illustrate similarity of voltage-dependence. D. Exemplar *I_Ca_* traces acquired at +10 mV from control (black) and *dr_Rem2* (red) expressing neurons. Traces are depicted without (left) and with normalization (right) to maximal current. Dotted line represents zero current level.

Ca^2+^ currents were evoked with a 70 ms test pulse to various potentials from a holding potential of −80 mV in solutions (see Methods) designed to eliminate current components arising from other voltage-gated ion channels (e.g., primarily Na^+^ and K^+^). Under control conditions (neurons heterologously expressing EGFP only), inward currents became apparent around −30 mV, reached a maximum around +10 mV, and thereafter declined reaching a zero current asymptote near +80 mV (Fig. 1A; open circles). Current density (pA/pF) was determined from the *I_Ca_* amplitude measured isochronally at 10 ms after initiation of the test pulse divided by the membrane capacitance (*C_m_*) as determined from integration and fitting the decay of a capacitive transient (see Methods). In neurons previously injected with *Danio rerio* cDNA (100 ng/µl) encoding RGK protein orthologs, the mean *I_Ca_* density was markedly reduced across a broad range of voltages (Figure 1A, filled symbols). Mean *I_Ca_* density at +10 mV (maximal current) was reduced by 83–97% by expressing RGK proteins (Fig 1B). The mean *C_m_* for RGK protein expressing neurons ranged from 42–53 pF. None of the values were significantly different from the *C_m_* of the control group (mean 45 pF; not shown) indicating current density reductions arose from effects on channels rather than plasma membrane surface area.

Normalization of the *I-V* curve (Fig 1A) to the maximal *I_Ca_* density (+10 mV) revealed that expression of RGK proteins appeared to reduce current density by a constant proportion at each voltage. An exemplar of *dr_Rem2* vs. control normalized *I-V* curves is illustrated in Fig. 1C. Despite a reduction in mean current density of 93% by *dr_Rem2*, the normalized *I-V* curves come close to superimposing over the entire voltage range. Individual current traces for *dr_Rem2* expressing neurons appeared similar when scaled to control currents (Fig. 1D) as previously shown for mammalian Rem2 expression [12] indicating no overt changes in activation or inactivation kinetics. However, this was not studied in detail as the large suppression of current amplitude in most cases made such comparisons difficult to interpret.

### Cloning and expression of Drosophila melanogaster RGK orthologs

The presence of RGK protein homologs in the protostome phyla (e.g., arthropods, nematodes, mollusks, etc.) has not been reported to our knowledge. BLAST searches of the *Drosophila melanogaster* genomic database using human RGK proteins as the source revealed three potential isoforms annotated as RGK1 (AAF57577), RGK2 (AAF57577), and RGK3 (ABV53867). Given the evolutionary distances involved, it was difficult to ascertain whether the sequences represent true orthologs with a 1∶1 relationship with vertebrate RGK protein subtypes. Hence, for non-vertebrate protein sequences, we will use the term RGK-like protein homolog. RGK-like protein homolog open reading frames were cloned from *Drosophila melanogaster* polyA RNA following reverse transcription and PCR amplification. The clone, denoted *dm_RGK1*, has an open reading frame predicting a 498 amino acid protein identical to AAF57577 except for a single Q116E substitution. We were unsuccessful at generating a full length RGK2 PCR product based on the nucleotide sequence for AAF57577. However, amplification from the second methionine (M165) was successful and the predicted product matched residues 165–740 of AAF57577 which we denote as *dm_RGK2t*. The (predicted) truncated product contains the entire Ras-like G-domain and carboxyl-terminus elements previously shown necessary for Ca^2+^ channel modulation [12]. Cloning of RGK3 resulted in 2 clones: 1) *dm_RGK3* (identical to ABV53867) and 2) *dm_RGK3L* which contains a 29 residue insertion starting after residue 401Q and is apparently a variant resulting from alternative splicing. Comparison of *Drosophila melanogaster* and *Homo sapiens* RGK G-domains revealed a 47–56% identity and conservation in the last seven amino acids previously identified as the C-7 motif [37]. The later is significant in that the C-7 motif appears specific to RGK proteins. In contrast, the N-termini of *Drosophila melanogaster* RGK proteins are longer (*ca*. 250–500 residues) than vertebrate N-termini (*ca.* 80–150 residues) and share little apparent sequence similarity.

**Figure 2:**
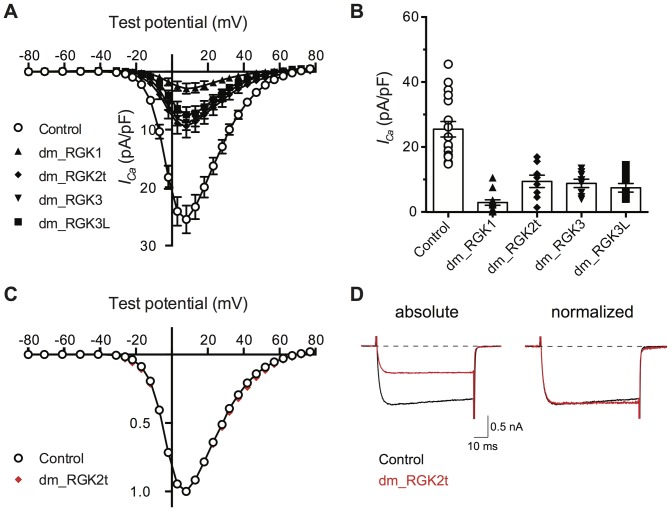
Fruit fly RGK-like protein homologs reduce *I_Ca_* density in rat sympathetic neurons. A. *I-V* plots in which mean ± SEM *I_Ca_* density is plotted versus test potential. *I_Ca_* was evoked and acquired as described for Fig. 1. Control neurons (open circles) were not injected with cDNA (n = 17). Neurons previously injected with *Drosophila melanogaster* RGK protein cDNA clones (50 ng/µl approximately 18–24 hours prior to recording) are depicted with filled symbols: *dm_RGK1* (triangle, n = 13); *dm_ RGK2t* (diamond, n = 8), *dm_RGK3* (inverted triangle, n = 8), and *dm_RGK3L* (square, n = 9). B. Category plot of data shown in panel A for *I_Ca_* density at +10 mV. Mean *I_Ca_* density for neurons expressing RGK protein clones differed significantly (*P*<0.05) from control (one-way ANOVA followed by Dunnett's multiple comparisons test). C. Normalized *I-V* plots for control (open circle) and *dm_RKG2t* expressing (red filled diamond) neurons. Data, from panel A, was normalized to the maximal *I_Ca_* density and plotted to illustrate similarity of voltage-dependence. D. Exemplar *I_Ca_* traces acquired at +10 mV from control (black) and *dm_RGK2t* (red) expressing neurons. Traces are depicted without (left) and with normalization (right) to maximal current. Dotted line represents zero current level.

To assess function, the *Drosophila melanogaster* RGK clones were expressed in rat SCG neurons and whole-cell patch-clamp recordings of *I_Ca_* performed (Fig. 2) as described above for the *Danio rerio* clones. Similar to vertebrate RGK protein clones, expression of all *Drosophila melanogaster* RGK-like protein homolog clones produced a decrease in sympathetic neuron *I_Ca_* density while producing little overt alteration in the shape of the *I-V* curve or individual current traces. Attenuation of peak current density (pA/pF) ranged from 63–86% (Fig. 2B) while mean *C_m_* was not significantly altered (mean *C_m_* ranged from 50–59 pF compared with a control of 56 pF). As an exemplar, the *dm_RGK2t I-V* curve was normalized to the control *I-V* curve (in this case, uninjected SCG neurons) as illustrated in Fig. 2C. The near superimposition of the curves demonstrates that mean current density was similarly attenuated over a wide range of test potentials. Likewise, normalization of individual currents traces (Fig. 2D) showed little change in current kinetics suggesting activation and inactivation were not greatly altered by *dm_RGK2t* expression.

To examine whether expression of other Ras subfamily members affected SCG neuron *I_Ca_* density, experiments were performed using human Rit1, Rit2, and diRas2. Mammalian Rit1 and Rit2 often showed up in BLAST search hit lists with scores similar to protostome RGK-like protein homologs. DiRas2 was examined because of similarities in the region distal to the G5 motif (α5-helix, see below). Following cDNA injection into rat SCG neurons, none of these constructs significantly reduced mean *I_Ca_* density when compared with uninjected control neurons (Figure S2). Hence, the ability of RGK-like proteins to attenuate *I_Ca_* density following expression appears specific, at least within this experimental context.

Taken together, the expression of RGK protein orthologs from *Danio rerio* and homologs from *Drosophila melanogaster* in rat sympathetic neurons recapitulates, at least superficially, the *I_Ca_* phenotype resulting from rat Rem2 heterologous expression [12]. Although the precise mechanism of Ca^2+^ channel inhibition by these RGK orthologs is unclear, the conservation of phenotype from diverse and evolutionarily distant species strengthens interpretations based on sequence comparisons.

### Identification of RGK orthologs from diverse organisms

Protein BLAST searches [30] using human RGK proteins as the search query accurately identified vertebrate orthologs. The search for more diverse sequences related to RGK proteins became increasingly complex as more evolutionarily distant genomes were examined. As search stringency decreased, other members of the Ras superfamily appeared in the hit lists (Rit1 and Rit2 being the most common contaminants) due to the homology of the Ras G-domain [38], [39]. In addition to vertebrate orthologs, our earlier searches revealed potential deuterostome RGK-like protein homologs in the sea lancet, *Branchiostoma floridae* (XP_002594243), and the purple sea urchin, *Strongylocentrotus purpuratus* (XP_785320). In addition to the *Drosophila melanogaster* RGK-like protein homologs, numerous protostome sequences were apparent in a large number of insect species (e.g., fruit flies, ants, bees, etc.) with sequenced genomes. We also found partial arthropod sequences from the wood tick, *Ixodes scapularis* (XP_002402890) and the water flea, *Daphnia pulex* (EFX75065). From these sequences it became apparent that, in addition to the G-domain, the last eleven residues, encompassing the C-7 domain [37] were highly conserved. Using alignment of the last eleven residues, we developed a search pattern (see Methods) to use with Pattern Hit Initiated BLAST [31], PHI-BLAST, and a human RGK protein sequence as search query (Gem or Rad). When followed by a second iteration of Position-Specific Iterated BLAST (PSI-BLAST), the hit list contained a large number of potential protostome RGK-like protein homologs that did not appear in previous searches. Multiple putative homologs for the sea hare, *Aplysia californica,* the pacific oyster, *Crassostrea gigas,* and the polychaete worm, *Capitella teleta,* were identified. It should be noted that many of the sequences are predicted (denoted XP, predicted protein model) from the genomic sequencing data and require further validation. Together with representative vertebrate sequences, the G- and C-terminus domains were aligned using the ClustalW algorithm.

### Alignment of the RGK protein G-domain

**Figure 3:**
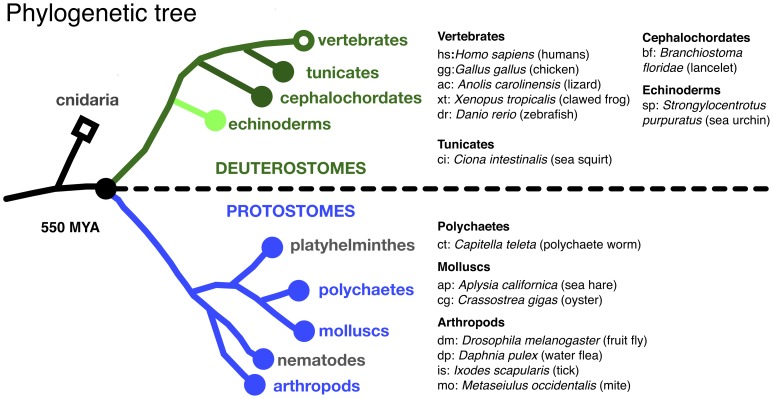
Phylogenetic tree for RGK protein orthologs. Phylogenetic tree layout was adapted from Yau and Hardie [40]. The deuterostome branch is labeled in green with dark green representing the phylum chordata. Tunicates and cephalochordates are subdivisions within this phylum with vertebrates representing the major group. Echinoderms (e.g., starfish, sea urchins, sea cucumbers) represent the second largest grouping of deuterostomes and are labeled in light green. The protostome branch is depicted below the dotted line in blue. Major phyla for which RGK protein orthologs/homologs were identified are presented in blue and the others in gray. Organisms with two letter abbreviation and common name (in parentheses) are depicted on the right.

**Figure 4:**
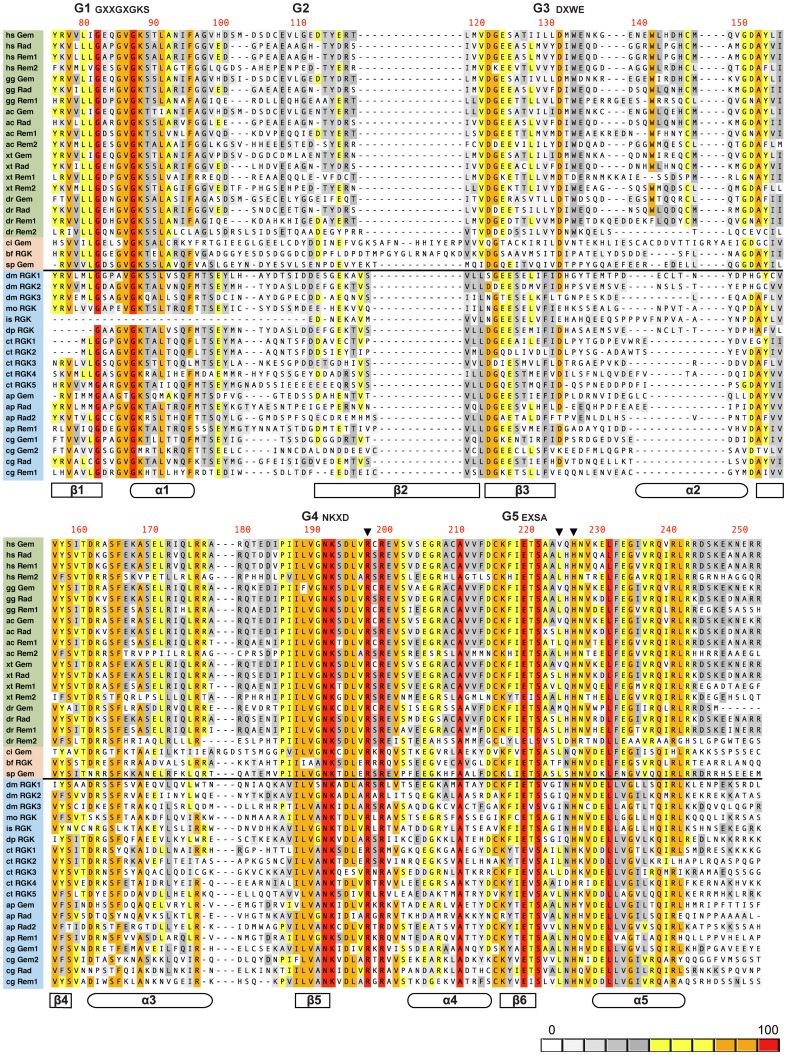
Alignment of RGK protein G-domains. G-domain sequences were aligned using the ClustalW algorithm in MacVector (version 12.7.5). Sequence labels (left) are color coded with vertebrates in green, non-vertebrate deuterostomes in pink, and protostomes in blue. The black horizontal line divides the deuterostomes/protostome sequences. Organism genus and species abbreviations (e.g., hs for *Homo sapiens*) are depicted in Figure 3. The method for parsing the RGK protein G-domain sequence is documented in the text and supplement (table S1) along with GenBank accession numbers for each sequence. The G-motifs (G1–5) along with canonical RGK protein sequences for G1, G3, G4, and G5 are depicted above the alignments. The residue numbers are for human Gem (top row) and are right justified. The inverted black triangles represent key residues participating in RGK protein–Ca_v_β interaction[43]. Secondary structural domains (α-helices and β-sheets) are depicted schematically below the sequences. Sequence identity color scale is depicted in the lower right corner.

A phylogenetic tree [40] with annotated representative species from which RGK protein orthologs and homologs were aligned is shown in Figure 3. Alignment of the RGK G-domain, color-coded for sequence identity, is shown in Figure 4. Unlike most Ras superfamily proteins, RGK proteins have extended N-termini preceding the G-domain. Although generally about 60–80 residues in vertebrates, the N-termini length in protostome homologs varied greatly from being potentially absent (ELU18455, *Capitella teleta*) to over 500 residues (e.g., AAF57675, *Drosophila melanogaster*). It should be noted, however, that many of the predicted transcripts have not been verified nor have protein products been detected. To identify the putative RGK G-domains, searches were performed on the highly conserved G-motifs, G1 (GXXGXGKS) and G5 (EXSA). G-motifs (G1–5) are canonical sequences involved in binding of GDP/GTP and Mg^2+^ found in both small-GTPase and heterotrimeric G-proteins. The start of the G-domain was defined as -6 residues from the start of G1 which encompasses the first β-sheet (β1) defined in structural studies of Ras superfamily proteins [41]. The end of the G-domain was defined as +30 residues from the beginning of the G5 motif, which includes the last α-helix (α5) in the structures, plus about 10 additional residues. The G1 and G5 motifs were less conserved in protostome and non-vertebrate deuterostomes with variations as noted in the supplement (Table S1). G-domains, determined in this manner, were between 170–180 residues with the exception of non-vertebrate deuterostomes *Branchiostoma floridae* (XP_002594243) and *Ciona intestinalis* (XP_002128442) which had insertions in the predicted G-domain. The alignment is composed of representative species with vertebrates comprising about half the sequences and non-vertebrate deuterostomes and protostomes comprising the remainder.

Examination of the alignment shows strong conservation in the G1, G4, and G5-motifs as well as sequences corresponding to the β4, α3, and β5 structural elements (Fig. S1B). As previously noted [9], the G2-motif in RGK proteins is poorly conserved compared with nearly all Ras superfamily members [38], [39] and this becomes more evident when evolutionarily diverse RGK-like protein family members are considered. As this region in RAS re-orients during GDP/GTP exchange (switch I region) and provides a major effector binding surface, the lack of conservation in RGK orthologs is consistent with structural studies indicating lack of order in this region and the current notion that RGK proteins deviate from the canonical GDP/GTP switch mechanism. The G3 motif in RGK proteins, DXWE, has been noted to differ from the highly conserved DXXG motif found in most Ras superfamily members [39], and has been proposed as an RGK protein signature sequence based on vertebrate sequences [41]. However, the G3 motif sequence conservation diminishes in non-vertebrate deuterostome and all protostome sequences. A similar pattern is noted for the W141 (residue numbering for hs Gem) and cysteine residue (C146) in the α2 helix that are highly conserved in vertebrate RGK proteins but missing from other sequences. Conversely, the G4 and G5 motifs, regions involved in binding with the guanine base, show a high degree of conservation that extends into non-vertebrate species (see also Fig. S1A). Several of the key residues in G4 (N191, K192) and G5 (E219, S221) show absolute conservation across all the species examined. Finally, the region distal to the G5 motif comprising the β6–α5 linker and α5 helix is not particularly well conserved among Ras subfamily members [38] yet shows a high degree of conservation in RGK-related proteins (Fig. S1B). Interestingly, a previous study [42] implicates this region of Gem for both binding to Ca_V_β subunits and producing Ca^2+^ channel inhibition.

Interaction of RGK proteins with the Ca_V_β subunits [7] is proposed to be integral to channel inhibitory function although alternative mechanisms have been put forth [16]. A comprehensive alanine-scanning mutagenesis study [43] based on Gem and Ca_v_β3 interaction assayed with immunoprecipitation revealed three residues in the mouse Gem G4–G5 domain region (R196, V223, and H225) that influenced this interaction without interfering with GDP/GTP binding. Interestingly, these residues are not conserved within the Ras superfamily and are thus potentially specific to RGK function. Sequence comparison across diverse species (residues indicated by inverted black triangles in Fig. 4) strongly supports the mutagenesis results. Residues cognate to mouse Gem R196 and H225 are nearly 100% conserved for all species examined. The V223 residue is less conserved with valine or leucine accounting for most of the deuterostome residues. In some protostome RGK-like protein homologs, including *Drosophila melanogaster*, the cognate residue is an isoleucine and, as shown earlier, expression of these RGK-like proteins are capable of inhibiting Ca^2+^ channel function. Isoleucine was one of several hydrophobic residues that maintained Gem-Ca_V_β interaction when substituted at this site [43]. In a complementary fashion, the Ca_V_β residues with which R196, V223, and H225 putatively interact are very highly conserved (rat Ca_V_β3 D194, D270, D272). The Ca_V_β conservation may point to conservation of the RGK-Ca_V_β interface, although the Ca_V_β GuK domain, in general, is very highly conserved. Nonetheless, the conservation of the RGK unique residues in both protostomes and deuterostomes argues strongly for the RGK protein-Ca_V_β interaction comprising a major function of this class of proteins.

### Alignment of the RGK protein carboxyl terminus

**Figure 5:**
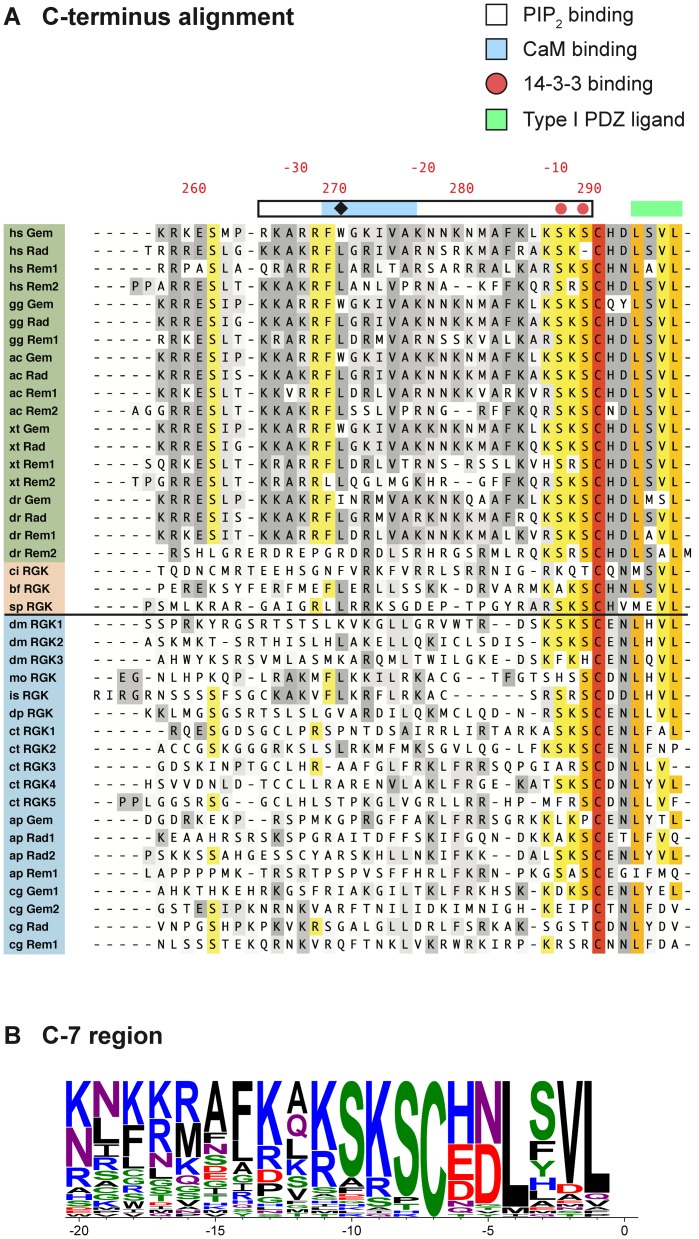
Alignment of RGK protein C-termini. A. The last 40 residues of RGK and RGK-like protein sequences were aligned using the ClustalW algorithm in MacVector (version 12.7.5). Sequence labels and identity color-coding are as shown in Figure 4. Previously defined functional domains identified in vertebrate RGK proteins are depicted schematically above the sequences. Residue labeling is for *hs* Gem (lower numbering) or in residues from the stop codon (upper numbering). B. Sequence logo depiction of the terminal 20 amino acids including the RGK protein signature C-7 domain. Web logo was created with WebLogo 3 (http://weblogo.threeplusone.com) [51].

The last 40 residues of RGK and RGK-like proteins were aligned using ClustalW (Fig. 5A). Among vertebrate RGK proteins, several motifs have been documented in this region. A Ca^2+^/calmodulin binding site [44] and polybasic region are located between residues -35 and -15 (relative to the stop codon). The latter is involved in plasma membrane targeting presumably via binding to anionic phospholipids such as PIP_2_ [45]. A serine residue at -8, when phosphorylated, is believed to be a 14-3-3 protein binding site. The most intriguing element is the C-7 motif [37] which starts with a highly conserved cysteine residue and continues to the end of the protein (Fig. 5B). The function of this motif is unclear but it appears to be unique to RGK proteins. Although cysteine residues in the C-termini of many Ras superfamily proteins are often prenylated (e.g., the canonical CAAX box), evidence for post-translational modification of C-7 is lacking. Finally, the last four residues of most vertebrate RGK proteins comprise a potential class I PDZ ligand [46], X-S/T-X-V/L. At present, PDZ domain containing proteins have not been identified that interact with RGK proteins.

Examination of the carboxyl-terminus alignment reveals that overall conservation noted previously for vertebrate orthologs decreases in more diverse organisms. Even among vertebrate RGK proteins, *Danio rerio* Rem2 shows an apparent deviation in the polybasic and calmodulin binding regions. As expression of *Danio rerio* Rem2 as well as *Drosophila melanogaster* RGK-like protein homologs decreased *I_Ca_* density in SCG neurons, these residues are not essential to this phenotype. Conversely, the C-7 motif plus 3–4 additional residues are highly conserved in all sequences. In particular, the cysteine residue is invariant in all orthologs/homologs examined suggesting a highly conserved but unknown function. The S/T and basic residues immediately preceding the cysteine are also well conserved suggesting that 14-3-3 protein binding may be of ancient origin. The residue at -6 is well conserved in both deuterostomes and protostomes but is a histidine in the former and an acidic residue (D/E) in the latter. The leucine at -1 and -4 is moderately well conserved in RGK orthologs. However, the S/T at -3, a residue critical to the Type PDZ ligand motif, is not conserved in protostomes where it tends to be an aromatic residue. Taken together, these data show that the last ten residues of RGK and related proteins are highly conserved and comprise a signature unique to RGK proteins that is not found in other Ras superfamily members (Fig. S1C).

## Discussion

Here we provide evidence for RGK-like protein homologs in the genomes of non-vertebrate deuterostomes and protostomes suggesting that a common RGK protein ancestor arose prior to the split between deuterostomes and protostomes approximately 550 million years ago. Moreover, expression of cloned RGK protein ortholog/homolog open reading frames from *Danio rerio* and *Drosophila melanogaster* mRNA in mammalian neurons decreased *I_Ca_* density establishing conservation of this phenotype. Sequence comparisons across evolutionarily diverse organisms revealed conserved residues unique to RGK-like proteins previously implicated in Ca^2+^ channel function. Taken together, the data suggest that interaction between RGK protein orthologs/homologs and voltage-gated Ca^2+^ channels arose during the earliest stages of differentiation from the Ras superfamily of proteins.

Two interesting observations arose during cloning of RGK protein related sequences. First, prior to searching the *Danio rerio* genome, we anticipated identifying duplicates of RGK protein orthologs. Teleost fish, such as *Danio rerio*, have undergone an additional cycle of whole-genome duplication compared with other vertebrates and hence often possess gene duplicates, termed ohnologues, that resulted from this expansion [47], [48]. For example, several Ca_V_β subunits (Ca_V_β2, Ca_V_β3, and Ca_V_β4) are both duplicated and expressed in zebrafish [49]. The absence of apparent RGK protein ohnologues in *Danio rerio* led us to speculate that high RGK protein gene dosages were not tolerated and thus duplicates heavily selected against. However, ohnologues of Rem2 were identified in the rainbow trout, *Oncorhynchus mykiss*, [50] weakening this notion. Second, cloning of *Drosophila melanogaster* RGK3 revealed a novel splice variant that was active in reducing Ca^2+^ channel density. To our knowledge, this is the first RGK-like protein splice variant cloned from cDNA shown to have effects on Ca^2+^ channels. The extent and significance of sequence diversification resulting from alternative splicing of RGK proteins is unclear.

The reduction of *I_Ca_* density in sympathetic neurons following expression of *Danio rerio* and *Drosophila melanogaster* RGK orthologs/homologs closely resembled the phenotype observed when mammalian Rem2 was overexpressed [12]. Thus, these RGK protein relatives might act similarly to reduce current without affecting channel surface density. It is also possible that channel endocytosis, in this case, could be enhanced as suggested previously [15] as we did not directly measure Ca^2+^ channel surface density. Interruption of Ca^2+^ channel trafficking to the plasma membrane by RGK proteins is less likely in the adult sympathetic neuron assay given the slow turnover of Ca^2+^ channels [12]. However, disruption of forward Ca^2+^ channel trafficking by vertebrate RGK proteins has been postulated and thus protostome homologs may share this ability as well.

The role that RGK protein orthologs/homologs play in *Danio rerio* and *Drosophila melanogaster* Ca^2+^ channel physiology is unclear although both organisms contain voltage-gated Ca^2+^ channel genes similar to those found in mammals. Of note, Ca_V_β sequences are conserved in both organisms. The genetic malleability of *Danio rerio* and *Drosophila melanogaster* should provide opportunities to further explore this question. Currently, the function of RGK-like proteins in protostomes is unknown.

**Figure 6:**
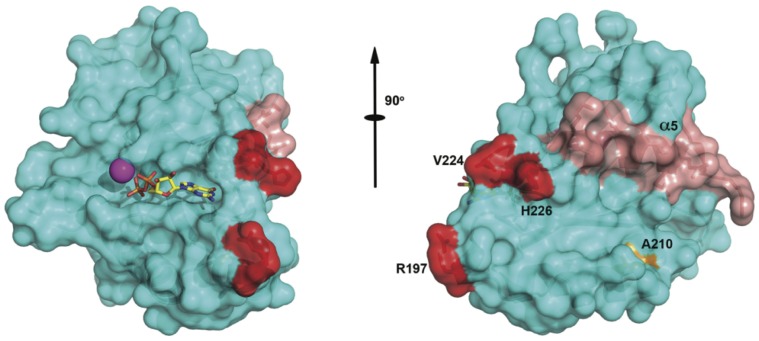
Location of RGK protein structural determinants. Shown are two perspectives, related by a 90° rotation along the drawn axis, of the RGK G-domain in surface representation. Secondary structure is depicted along with labeled residues underneath the surface. Nucleotide (GDP) is shown in a bond representation and Mg^2+^ is seen as a magenta sphere. The red residues are the amino acids essential for RGK-Ca_V_ β association as denoted by inverted black triangles in Figure 4. Alpha helix 5 is shown in pink and the absolutely conserved Ala from alpha helix 4 is shown in orange. Residue numbering is based on *Homo sapiens* Gem (PDB code: 2HT6).

Using sequential PHI/PSI BLAST searches we identified numerous potential non-vertebrate RGK-like protein homologs not previously reported. In deuterostomes, single RGK-like protein homologs were identified in echinoderms, tunicates, and sea lancelets that differed significantly from vertebrate RGK proteins (Fig. 4). Whether these organisms contain additional RGK-like protein isoforms or contain only a single RGK-like protein is unclear. We also identified, for the first time, numerous potential RGK-like protein homolog sequences in protostomes. The available genomes are heavily represented by insects (flies, ants, bees, and wasps) and thus the majority of protostome homologs were found within these genomes. However we did identify potential RGK-like protein homologs in two mollusks, *Aplysia californica* and *Crassostrea gigas*, and a polychaete worm (*Capitella teleta*). Inclusion of representative protostomes to sequence alignments helped refine several concepts deduced from earlier RGK protein sequence comparisons. In terms of RGK proteins and Ca^2+^ channel function, three observations stand out. First, three residues identified in an alanine mutagenesis screen found to be important for Ca_v_β interaction with mouse Gem [43] are conserved in non-vertebrate RGK-like protein sequences. Significantly, these residues are not conserved within the greater Ras subfamily suggesting that RGK protein–Ca_v_β interaction is an ancient property that arose prior to the deuterostome/protostome split. Second, the sequence corresponding to the structural α5-helix shows a similar conservation pattern which is relatively specific to RGK proteins when compared to the greater Ras subfamily. Interestingly, this region has also been implicated in Ca^2+^ channel function [42]. These conserved G-domain residues, when mapped onto the Gem structure (Fig. 6) define a potential interaction surface important to the Ca^2+^ channel phenotype seen following RGK protein expression. Finally, the last eleven residues are highly conserved with a cysteine at position -7 being absolutely conserved in all sequences examined. The C-7 motif as a unique RGK protein signature sequence [37] and the addition of more diverse sequences strengthens this argument. We have used this sequence motif in PHI-BLAST searches to uncover additional potential RGK-like protein homologs in diverse organisms. At present, the function of this unique sequence is unclear.

Taken together, the data reveal the ancient origins of RGK proteins within the Ras superfamily. Sequence conservation and electrophysiological experiments suggest that RGK protein interaction with voltage-gated Ca^2+^ channels originated prior to the deuterostome/protostome split and that this function has been strictly conserved to present times.

## Supporting Information

Figure S1 **Sequence conservation of RGK protein orthologs/homologs.** Analysis of RGK and RGK-like protein sequences was performed using ConSurf [52]. Negative scores denote conserved amino acids. The multiple sequence alignments shown in Figures 4 and 5 were used as input. A. Conservation scores are drawn in histogram form for the RGK G-domain. Scores were smoothed with a moving average of five residues. Residue numbering is based on human Gem. The G-domain is depicted as a black strip with the labeled G loops in grey. B. Differential conservation scores between the Ras subfamily (67 different orthologs of H-Ras, K-Ras and M-Ras) and the RGK family (panel A), using the Gem G-domain residue numbering. No smoothing was employed. In this instance, positive values indicate greater differences in conservation between the two subfamilies. Notably, beginning with the G4 loop, RGK proteins are more conserved than the Ras counterpart. C. Conservation scores for the RGK orthologs in the C-terminal region.
(TIF)

Figure S2 **Heterologous expression of mammalian Ras family proteins with sequence homology to RGK proteins does not change** ***I_Ca_*** **density in rat sympathetic neurons.** A. Category plot for *I_Ca_* measured at +10 mV. Uninjected neurons (black circles, n = 14) were not injected with cDNA and recorded in parallel with injected neurons. Neurons previously injected with human Rit1 (AF084462, cloned into pcDNA3.1), human Rit2 (NM_002930, cloned into pcDNA3.1), mouse DiRas2 (NM_001024474, cloned into pCI-Kan) or Venus-tagged DiRas2 cDNA clones (50 ng/µl approximately 18–24 hours prior to recording) are depicted with filled circles: Rit1 (orange, n = 9); Rit2 (purple, n = 7); DiRas2 (red, n = 9), Venus-DiRas2 (green, n = 10). The mean *I_Ca_* for all injected groups was not significantly different (*P>*0.05) from uninjected controls (one-way ANOVA, Dunnett's multiple comparison test). B. Category plot for *I_Ca_* density measured at +10 mV from the same cells shown in A. The mean *I_Ca_* density for all injected groups ws\as not significantly different (*P>*0.05) from uninjected controls (one-way ANOVA, Dunnett's multiple comparison test). C. *I-V* plots in which mean ± SEM *I_Ca_* density (pA/pF) is plotted versus command potential (mV). *I_Ca_* was evoked and acquired as described for Fig. 1. D. Representative image of HeLa cells transfected with Venus-tagged DiRas2. HeLa cells (ATCC) were plated (2.0×10^4^ cells per ml) on poly-l-lysine coated glass bottom dishes (MatTek) in MEM +/+. Cells were transfected with 250 ng Venus-DiRas2 cDNA and 7 µl fully deacylated polyethylenimine (PEI) at 7.5 mM in 100 µl MEM-/- overnight. Cells were imaged the following day with a Retiga EXi 12-bit CCD camera (QImaging) mounted on a Zeiss Axiovert 200 inverted microscope using appropriate filters for Venus fluorescence and MicroManager software (v1.4.15). Scale bar is 100 µm.
(TIF)

Table S1 **RGK protein ortholog/homolog sequences used for alignments.** Protein sequence accession numbers are shown along with annotation. The sequence of the G1 motif is shown with deviations from the canonical sequence as illustrated. Residue numbers refer to the start and end of the G1 motif. The start of the G-domain is calculated at -6 from the start of the G1 motif. Similar information is depicted for the G5 motif with the G-domain end being calculated at +30 residues from the end of the G5 motif. The calculated length of the G-domain is shown (AA). The residue number for the conserved cysteine (C-7) is shown as well as the start and end of the C-terminus (last 40 residues were aligned).
(TIFF)
