# Supplementary figures and images for: Ancient Origins of RGK Protein Function: Modulation of Voltage-Gated Calcium Channels Preceded the Protostome and Deuterostome Split
Source: PLoS One. 2014 Jul 3;9(7):e100694. doi: 10.1371/journal.pone.0100694 (PMC4081519; doi:10.1371/journal.pone.0100694)

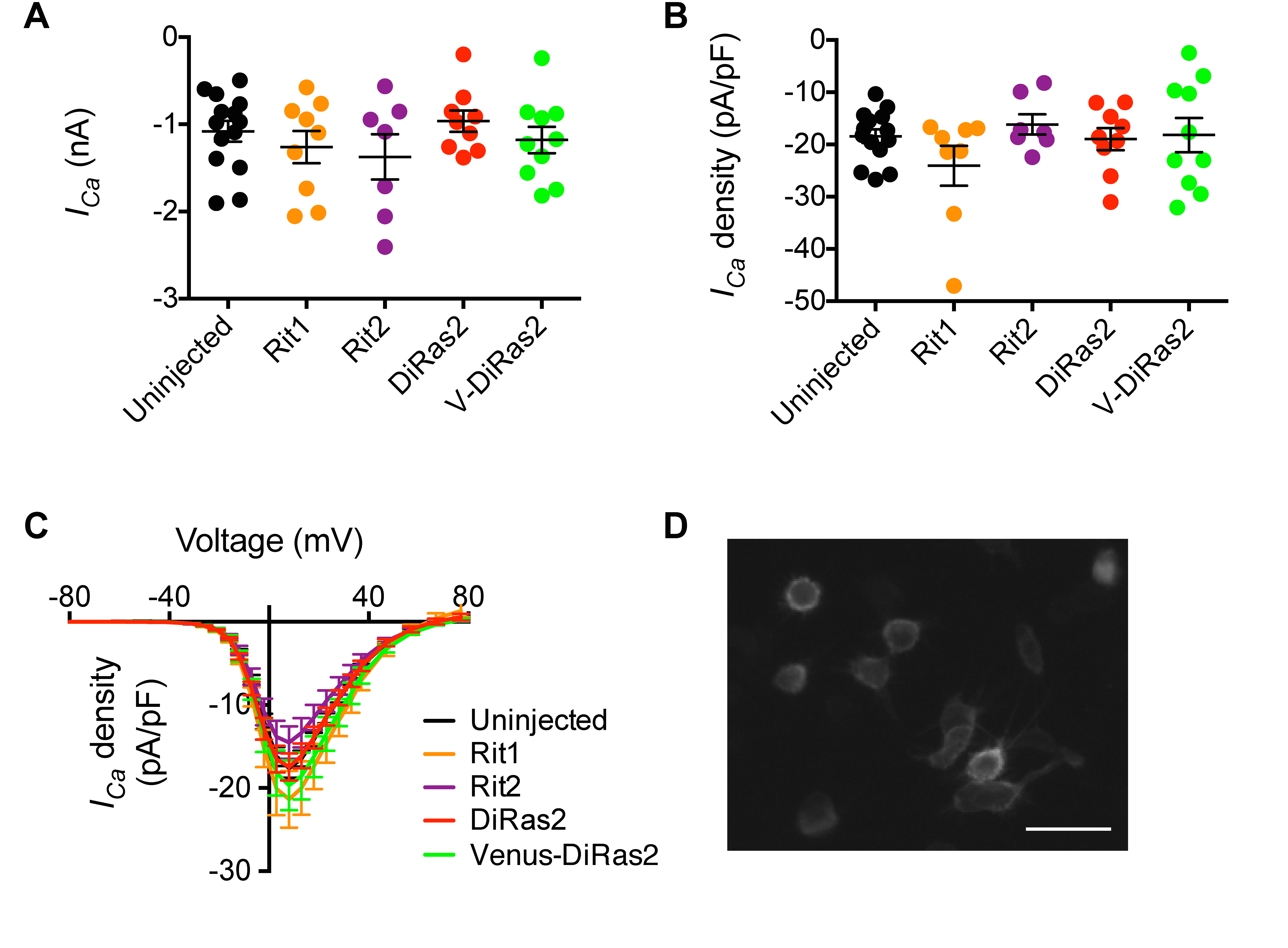

Supplement: Figure S2 — Heterologous expression of mammalian Ras family proteins with sequence homology to RGK proteins does not change ICa density in rat sympathetic neurons. A. Category plot for ICa measured at +10 mV. Uninjected neurons (black circles, n = 14) were not injected with cDNA and recorded in parallel with injected neurons. Neurons previously injected with human Rit1 (AF084462, cloned into pcDNA3.1), human Rit2 (NM_002930, cloned into pcDNA3.1), mouse DiRas2 (NM_001024474, cloned into pCI-Kan) or Venus-tagged DiRas2 cDNA clones (50 ng/µl approximately 18–24 hours prior to recording) are depicted with filled circles: Rit1 (orange, n = 9); Rit2 (purple, n = 7); DiRas2 (red, n = 9), Venus-DiRas2 (green, n = 10). The mean ICa for all injected groups was not significantly different (P>0.05) from uninjected controls (one-way ANOVA, Dunnett's multiple comparison test). B. Category plot for ICa density measured at +10 mV from the same cells shown in A. The mean ICa density for all injected groups ws\as not significantly different (P>0.05) from uninjected controls (one-way ANOVA, Dunnett's multiple comparison test). C. I-V plots in which mean ± SEM ICa density (pA/pF) is plotted versus command potential (mV). ICa was evoked and acquired as described for Fig. 1. D. Representative image of HeLa cells transfected with Venus-tagged DiRas2. HeLa cells (ATCC) were plated (2.0×10^4^ cells per ml) on poly-l-lysine coated glass bottom dishes (MatTek) in MEM +/+. Cells were transfected with 250 ng Venus-DiRas2 cDNA and 7 µl fully deacylated polyethylenimine (PEI) at 7.5 mM in 100 µl MEM-/- overnight. Cells were imaged the following day with a Retiga EXi 12-bit CCD camera (QImaging) mounted on a Zeiss Axiovert 200 inverted microscope using appropriate filters for Venus fluorescence and MicroManager software (v1.4.15). Scale bar is 100 µm. (TIF) [file pone.0100694.s002.tif]

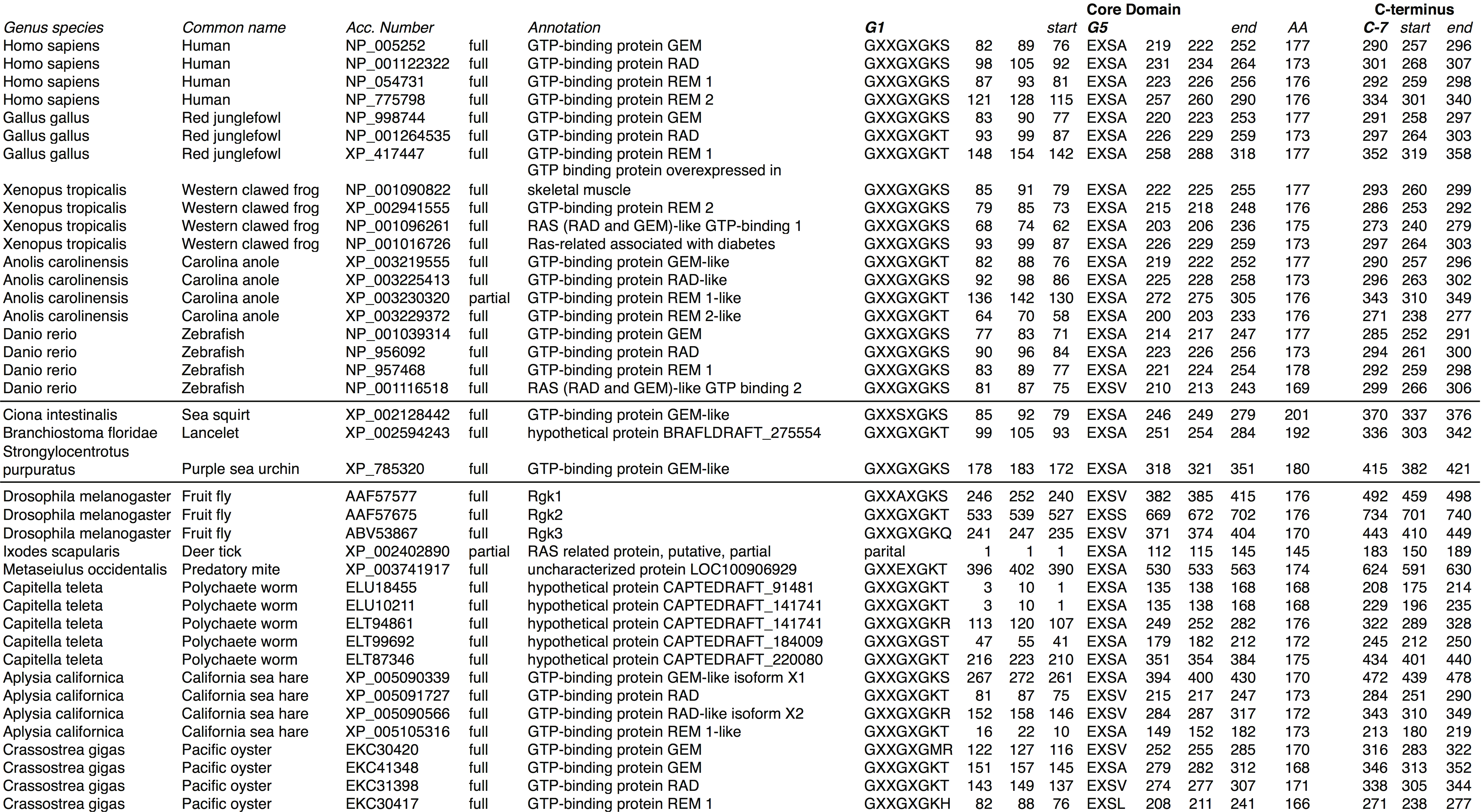

Supplement: Table S1 — RGK protein ortholog/homolog sequences used for alignments. Protein sequence accession numbers are shown along with annotation. The sequence of the G1 motif is shown with deviations from the canonical sequence as illustrated. Residue numbers refer to the start and end of the G1 motif. The start of the G-domain is calculated at -6 from the start of the G1 motif. Similar information is depicted for the G5 motif with the G-domain end being calculated at +30 residues from the end of the G5 motif. The calculated length of the G-domain is shown (AA). The residue number for the conserved cysteine (C-7) is shown as well as the start and end of the C-terminus (last 40 residues were aligned). (TIFF) [file pone.0100694.s003.tif]
